# The value of ^68^Ga-PSMA PET/CT in the diagnosis of intracapsular prostate cancer with a poor prognosis

**DOI:** 10.1007/s12672-024-01127-5

**Published:** 2024-07-02

**Authors:** Yajing Wang, Jieping Song, Lulu Yang, Wencheng Li, Wei Wang, Aiqing Ji, Liwei Wang, Feng Wang

**Affiliations:** 1 Department of Radiology, Nanjing First Hospital, Nanjing Medical University, 68Th Changle Road, Nanjing, 210006 China; 2 Department of Nuclear Medicine, Nanjing First Hospital, Nanjing Medical University, 68Th Changle Road, Nanjing, 210006 China; 3 Department of Pathology, Nanjing First Hospital, Nanjing Medical University, Nanjing, 210006 China; 4 Department of Urology, Nanjing First Hospital, Nanjing Medical University, Nanjing, 210006 China

**Keywords:** Prostate, Tumor, Positron-emission tomography, Prostate-specific membrane antigen

## Abstract

**Objective:**

To evaluate the diagnostic value of ^68^Ga-prostate-specific membrane antigen (PSMA) positron emission tomography/computed tomography (PET/CT) for intracapsular prostate cancer with a poor prognosis (PPC) and no extracapsular invasion or distant metastasis.

**Methods:**

The PET/CT images and clinical data of 221 patients were retrospectively analyzed. These patients all had clear pathological results. The maximum standard uptake value (SUVmax) of the main lesions was measured at the postprocessing workstation and was tested for correlation with the pathological score. The diagnostic accuracy was calculated using the receiver operating characteristic (ROC) curve, and the best diagnostic threshold was calculated. The correlation between SUVmax and the International Society of Urological Pathology Grade Group (GG) was also analyzed.

**Results:**

The pathological results of the 221 patients were 48 benign lesions and 173 malignant lesions, including 81 PPC. Low-, intermediate-, and high-risk prostate cancers made up 21.97% (38/173), 54.33% (94/173), and 23.70% (41/173) of the malignant lesions, respectively. SUVmax and GG were positively correlated (r = 0.54, P < 0.01). The best SUVmax thresholds for ^68^Ga-PSMA PET/CT for the diagnosis of intracapsular PC and PPC were 7.95 and 13.94, respectively; the specificities were 0.83 and 0.85, the negative predictive values were 0.55 and 0.87, and the areas under the ROC curves were 0.88 and 0.88, respectively.

**Conclusion:**

^68^Ga-PSMA PET/CT has high specificity and NPV in the diagnosis of intracapsular PPC, but the sensitivity for the diagnosis of intracapsular low-risk PC is low, which may cause some cases to be undetected.

## Introduction

Prostate cancer (PC) is the second most common malignant tumor in men worldwide (about 29%) [1]. With demographic aging and changes in people's lifestyles, PC morbidity and mortality are rising constantly. Therefore, accurate early detection and risk prediction are highly important for timely treatment and improvement of patient prognosis. This is especially true for intracapsular prostate cancer because when it spreads beyond the capsule or metastasizes, the treatment can become very ineffective. Studies are being performed on intermediate- to high-risk PC (IHPC), but IHPC also includes patients with grade group 2 (GG2). The risk level of these patients is usually lower than that of GG3 patients, and they are less likely to develop metastasis and recurrence. Therefore, we defined a poor prognosis prostate cancer (PPC) as that with GG ≥ 3, which has a significantly greater probability of metastasis and recurrence, a worse prognosis, and a lower 5-year survival rate than low-risk PC [2, 3]. PPC patients need more aggressive and longer-term radiation therapy and hormone therapy. Increasing the detection rate of intracapsular prostate cancer with poor prognosis before treatment helps patients receive the best treatment option in a timely manner and improves the survival rate. This topic still needs further study, so it is important to differentially diagnose them in clinical management.

Prostate-specific antigen (PSA) is often screened to diagnose PC, but its specificity is low, and it may be accompanied by overdiagnosis and overtreatment [4]. Transrectal ultrasound-guided biopsy (TRUS-GB) is the main method for the preoperative diagnosis of male prostate cancer with elevated serum PSA levels [5]. However, patients generally require hospitalization for examination, and missed diagnoses and complications can occur [6], such as bleeding, infection, etc. The pathological score of needle biopsy was inconsistent with the pathological score after radical resection. Imaging methods can help determine which males with elevated PSA continue to undergo biopsy, which may reduce unnecessary biopsies and improve diagnostic accuracy. Magnetic resonance imaging (MRI), a mature imaging method, is widely used in prostate examination [7] because the low signal density on T2-weighted images caused by stromal hyperplasia and chronic inflammation is similar to that on PC. Although its sensitivity is high, the specificity of MRI needs to be improved. Especially, small lesions and diseases within the central gland may not be easily identified by multiparameter MRI (mpMRI), and up to 35% of clinically significant prostate cancer may be invisible [8].

Prostate-specific membrane antigen (PSMA) is a type II transmembrane glycoprotein receptor found on the cell surface, and its expression is significantly elevated in more than 90% of prostate cancer cells (1000 times more than that in normal prostate cells). In recent years, targeted PSMA positron emission tomography/computed tomography (PET/CT) has shown relatively high value in the detection of PC metastasis and recurrent lesions [9–12]. As a new molecular analysis-based imaging method, it has higher specificity and sensitivity in detecting prostate tumor lesions and metastases compared to MRI. This application has been approved by the U.S. Food and Drug Administration, and its preliminary application has also begun in mainland China. PSMA PET/CT also has relatively high sensitivity and specificity in the diagnosis of primary PC, and its maximum standardized uptake value (SUV_max_) is an independent predictor of clinically significant PC [13].

In patients with high-risk scores and without metastatic disease, radical prostatectomy is the preferred treatment [14]. PC patients with metastasis often have a poor prognosis. The identification of PC patients with a poor prognosis before metastasis helps to guide treatment and improve their prognosis [15]. These patients can benefit the most from imaging. Lymph node and bone metastasis are prone to occur in PC patients with a poor prognosis, and qualitative diagnosis of the primary lesion is relatively easy [16]. Intracapsular PC is an early prostate cancer, and the prognosis of radical resection is good; therefore, early diagnosis is highly important. It is difficult to predict and diagnose intracapsular PC patients with no metastasis.

Such an approach is more clinically significant and can guide clinicians in timely and active treatment, but there is no relevant literature investigating this issue. This study evaluated the diagnostic value of ^68^Ga-PSMA PET/CT for identifying intracapsular PC patients with a poor prognosis who did not have extracapsular invasion or distant metastasis.

## Materials and methods

### Clinical data

**Fig. 1:**
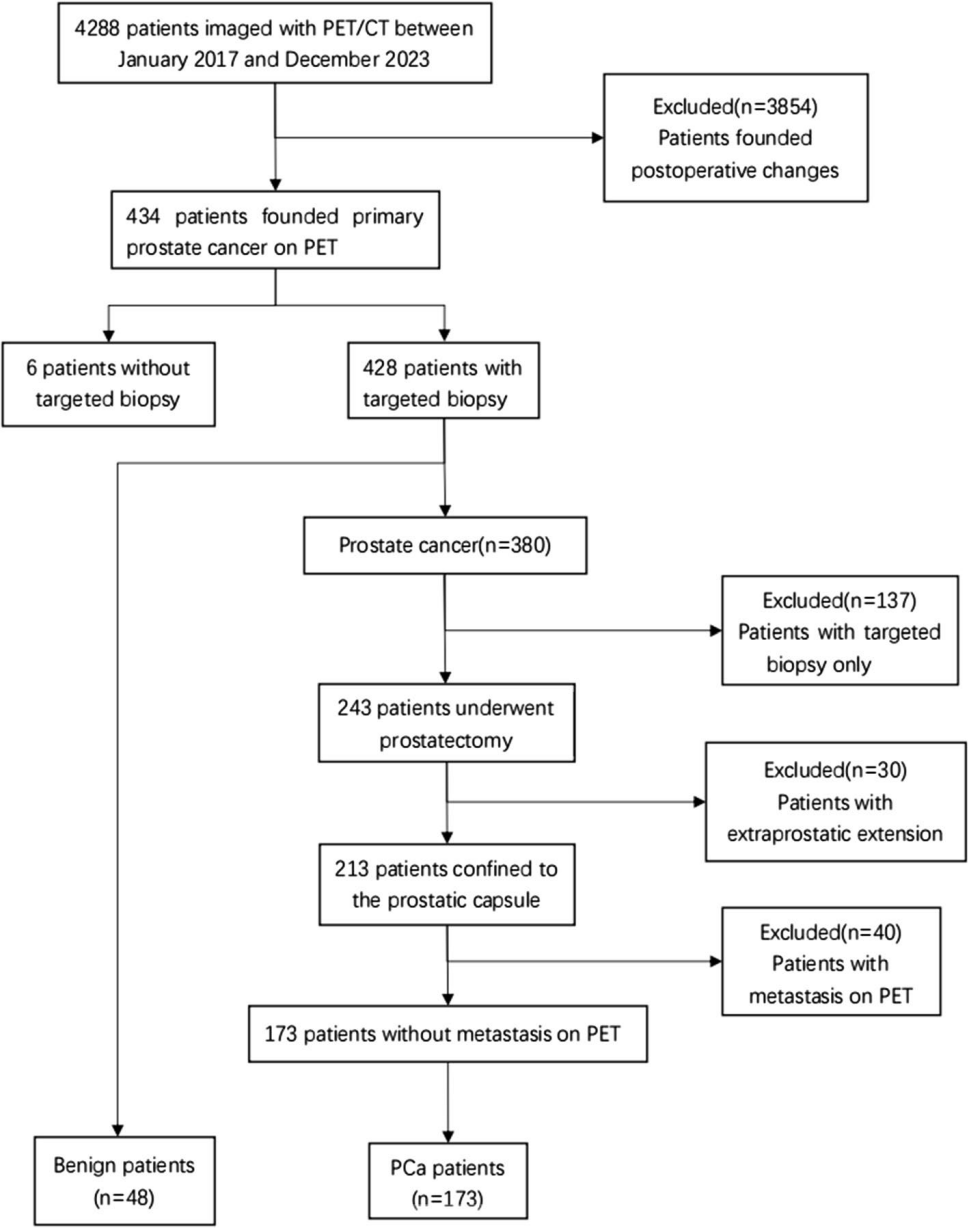
Flowchart of the included patients

The data of patients who underwent ^68^Ga-PSMA PET/CT at our hospital between January 2017 and December 2023 were retrospectively analyzed. Each patient's medical history included difficulty urinating, elevated PSA, hematuria, and low-back pain. The inclusion criteria for the study were as follows: (1) suspected prostate cancer at the first visit (elevated PSA level, positive MRI, or positive digital examination), (2) an interval between ^68^Ga-PSMA PET/CT and radical resection of no more than 60 days, and (3) needle biopsy and radical resection. The exclusion criteria were as follows: (1) the patient had received androgen deprivation therapy, radiotherapy, chemotherapy, or prostate surgery before PET/CT examination; (2) had an interval between radical resection and ^68^Ga-PSMA PET/CT examination more than 60 days; (3) had prostate cancer invasion into extracapsular tissues found on radical mastectomy; and (4) had lymph node, bone, or lung metastatic lesions found during PET/CT examination or radical mastectomy. A total of 221 consecutive patients were included in the study. The patient flowchart is shown in Fig. 1. All patients signed informed consent before ^68^Ga-PSMA PET/CT examination, and the Ethics Committee of Nanjing First Hospital approved this retrospective study.

### PET/CT examination and image analysis

^68^Ga-PSMA was synthesized in the class IV laboratory of our hospital using the automated labeling module of the International Thoracic Group (ITG), Germany. It was prepared following laboratory standards to radiochemical purity greater than 93%. ^68^Ga-PSMA was injected intravenously at an injection dose of approximately 2 MBq/kg. The patients were instructed to drink more water and urinate more often. The patient underwent spiral CT scan at approximately 45 min and PET scan (United Imaging, uMI780, Shanghai, China) at approximately 60 min. From the top of the cranium to the middle of the thigh, the PET data were collected from four beds, each bed taking approximately 3 min. The PET images and CT images were fused at the workstation to obtain multiplanar images, and the maximum standard uptake value was measured.

Before the pathological results were disclosed, two nuclear medicine physicians conducted blinded analyses of the PET images. The main diagnosis was based on the SUV_max_ of the main lesion. The main lesion was defined as that with the most obvious radioactive concentration. If no significant radioactive concentration was found in the prostate, then the area above background in the prostate with the highest SUV_max_ was selected as the target area of interest. The diameter of the measurement range was always greater than 1 cm. In this study, only lesions with a volume greater than 0.5 cm^3^ were included.

### Pathology

All patients underwent transrectal ultrasound-guided needle biopsy (TUNB). The malignant patients underwent radical prostatectomy. During TUNB, six punctures were taken on the prostate on each side, and the serial number of each puncture was recorded (12 in total). The score for benign lesions (BLs) was based on the puncture result, and the score for malignant patients after radical resection was used as the cutoff. Benign prostatic hyperplasia (BPH) and prostatitis belonged to the BL subgroup, patients with BL and GG1-2 belonged to the nonhigh-risk subgroup, and patients with GG ≥ 3 belonged to the poor prognosis subgroup.

### Statistical analysis

Normally distributed data are expressed as mean ± standard deviation, and the means were compared using Student’s *t* test. When the data did not fit a normal distribution, they are given as median (P_25_, P_75_), and the Mann‒Whitney U test compared their means. The relationships between the imaging and pathological results were assessed by Spearman correlation analysis. Statistical analysis was done with SPSS (version 25.0; IBM, Inc., Chicago, IL, USA) and GraphPad Prism (version 9.0; GraphPad Software, Inc., La Jolla, CA, USA). A receiver operating characteristic (ROC) curve was drawn and the area under the curve (AUC) calculated to determine the optimal SUV_max_ threshold. P < 0.05 indicated statistical significance.

## Results

**Fig. 2:**
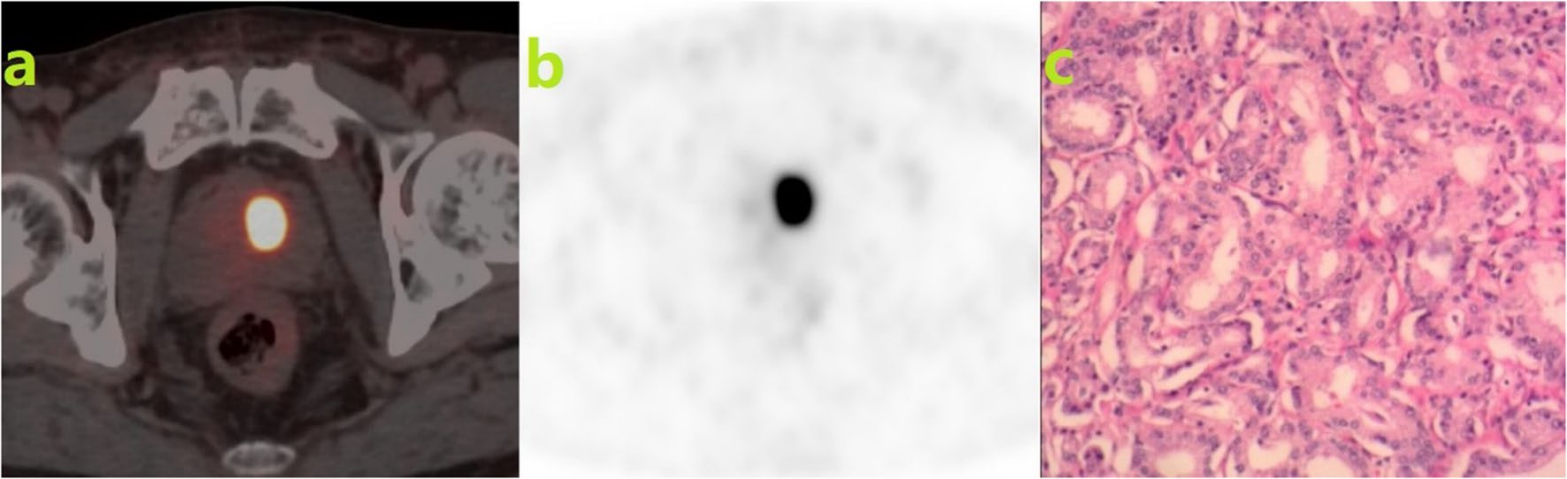
A 79-year-old male patient. Physical examination revealed elevated blood PSA (8.85 ng/ml). He did not usually have difficulty urinating. A nodular focus of higher radioactive uptake was observed in the left transition zone (**a**), with an SUV_max_ of 29.18 (**b**). Prostate cancer was confirmed by pathology: Gleason score: 3 + 4 = 7 (**c**)

Pathology revealed 48 benign lesions and 173 malignant lesions in 221 patients, including 81 cases with poor prognosis. The distribution proportions and SUV_max_ of the lesions are given in Table 1. Low-, intermediate-, and high-risk PCs made up 21.97% (38/173), 54.33% (94/173), and 23.70% (41/173) of the malignant lesions, respectively. The PET images of benign lesions showed very low radiotracer concentrations, similar to or slightly greater than those in normal tissues, though a few benign lesions had a moderate or greater radioactive concentration. The radiotracer concentration was obvious on the PET images of the PCs (Fig. 2), and the median SUV_max_ was greater than that of the benign tissues (P < 0.05). A very few malignant lesions did not have a significant radioactive concentration. The interval between radical treatment and PET/CT examination was 8 ± 4 days. SUV and GG did not correlate with the mCi value of the tracer (P > 0.05). SUV and GG were each correlated with age (P < 0.05). Table 2 compares the median SUV_max_ between groups.

**Table 1:** Results of the SUV _max_ of different groups

Groups	Cases (n = 221)	Median SUV_max_
Benign	48 (21.72%)	5.57 (4.29, 7.40)
Malignant	173 (78.28%)	13.68 (9.10, 21.90)
GG1	38 (21.97%)	7.15 (5.13, 10.25)
GG2	54 (31.21%)	12.29 (9.26, 16.15)
PPC	81 (46.82%)	20.86 (14.41, 28.23)
GG3	40 (23.12%)	22.01 (16.38, 25.98)
GG4	35 (20.23%)	20.19 (14.52, 30.42)
GG5	6 (3.47%)	18.34 (15.01, 23.26)

*PPC* poor prognosis prostate cancer, *SUVmax* maximum standardized uptake value

**Table 2:** Comparison of SUV_max_ between the groups

Groups	U	Z	P
Benign & Malignant	985	− 8.08	< 0.05
GG1 & Benign	590	− 2.80	< 0.05
GG1 & GG2	459	− 4.50	< 0.05
GG1 & GG3	116.5	− 6.43	< 0.05
GG2 & GG3	505	− 4.40	< 0.05
GG1 & PPC	210.5	− 7.57	< 0.05
GG2 & PPC	997	− 5.35	< 0.05

*PPC* poor prognosis prostate cancer, *SUVmax* maximum standardized uptake value

**Fig. 3:**
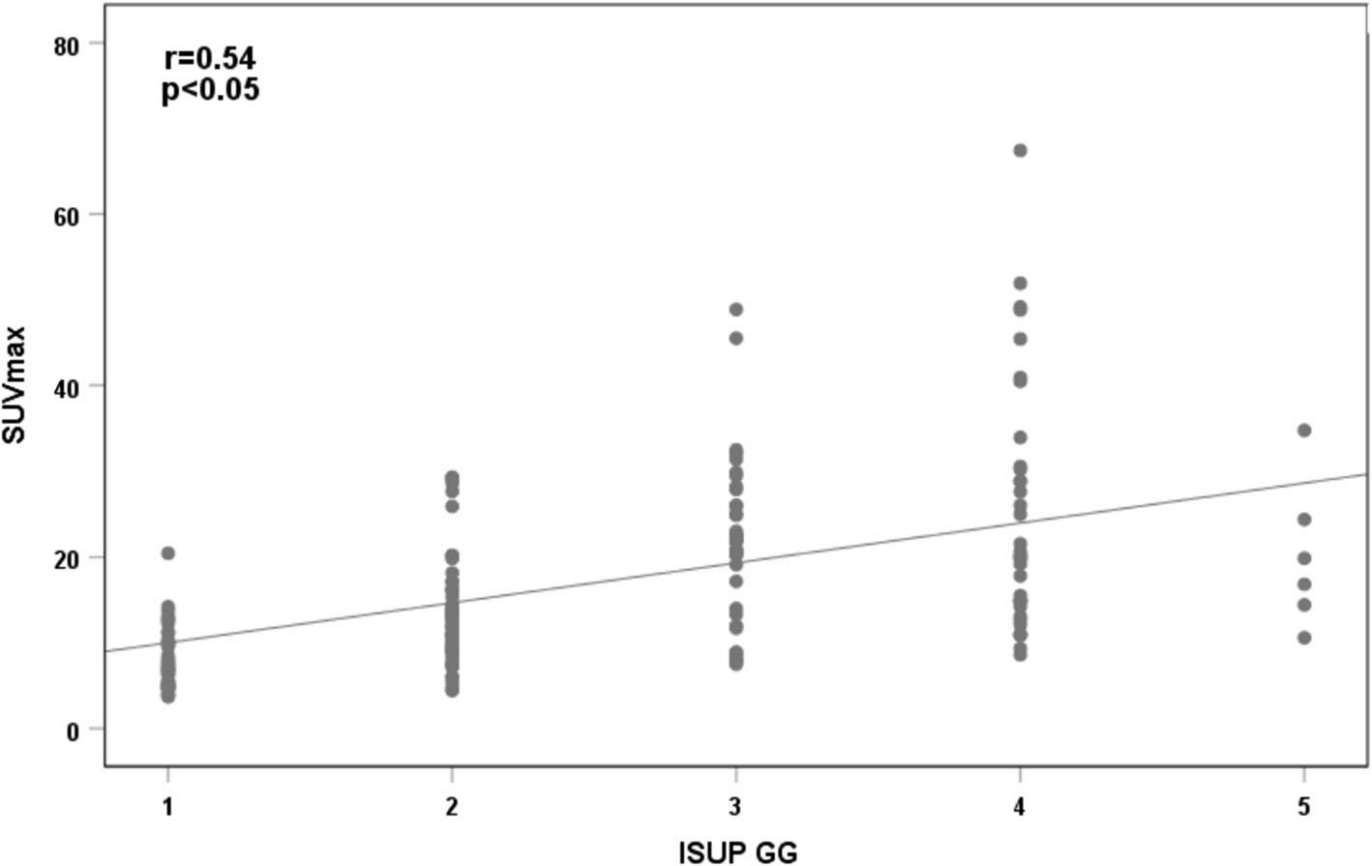
Scatter plot of the correlation between SUV_max_ and GG

**Fig. 4:**
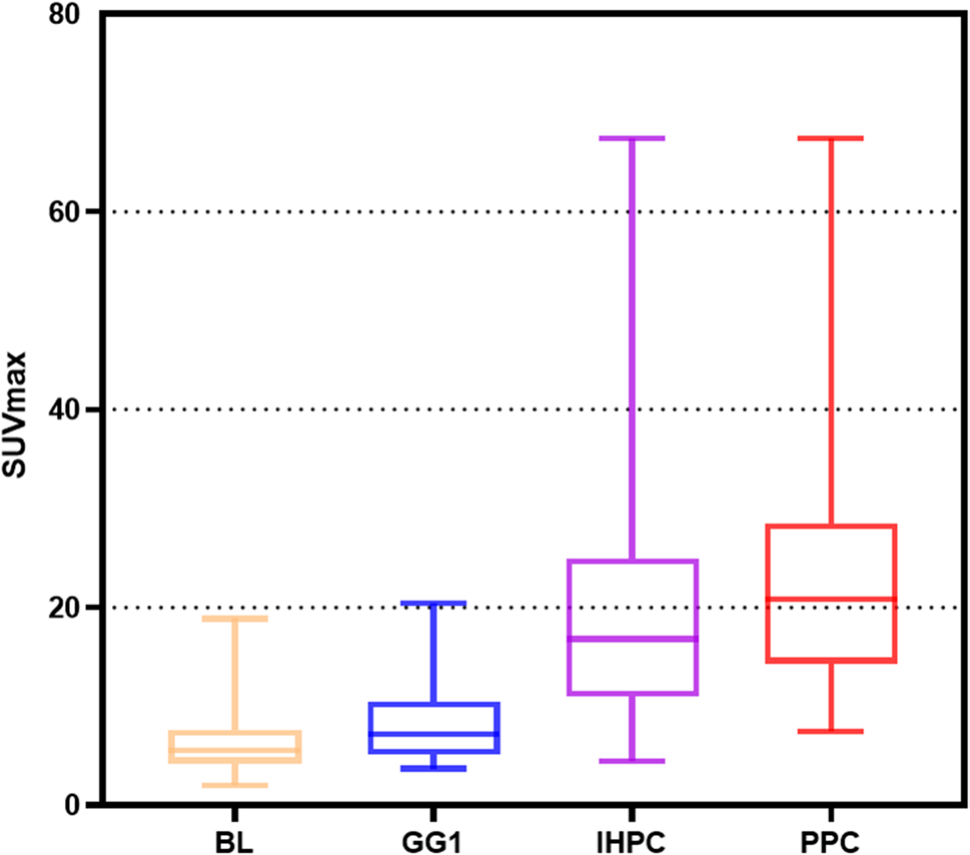
Box plot of SUV_max_ distribution in different risk groups

**Fig. 5:**
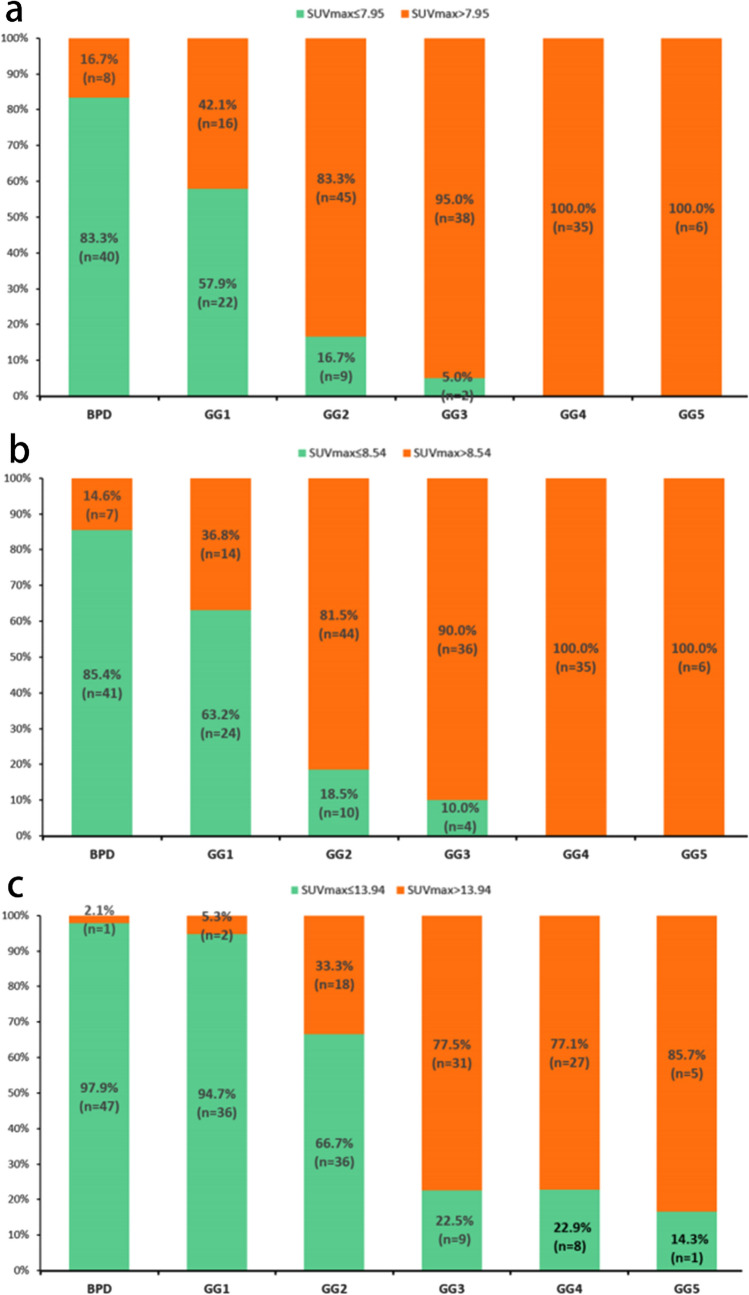
Percentage of patients in each group whose SUV_max_ was greater than the threshold:(**a**) PC (**b**) IHPC (**c**) PPC

**Fig. 6:**
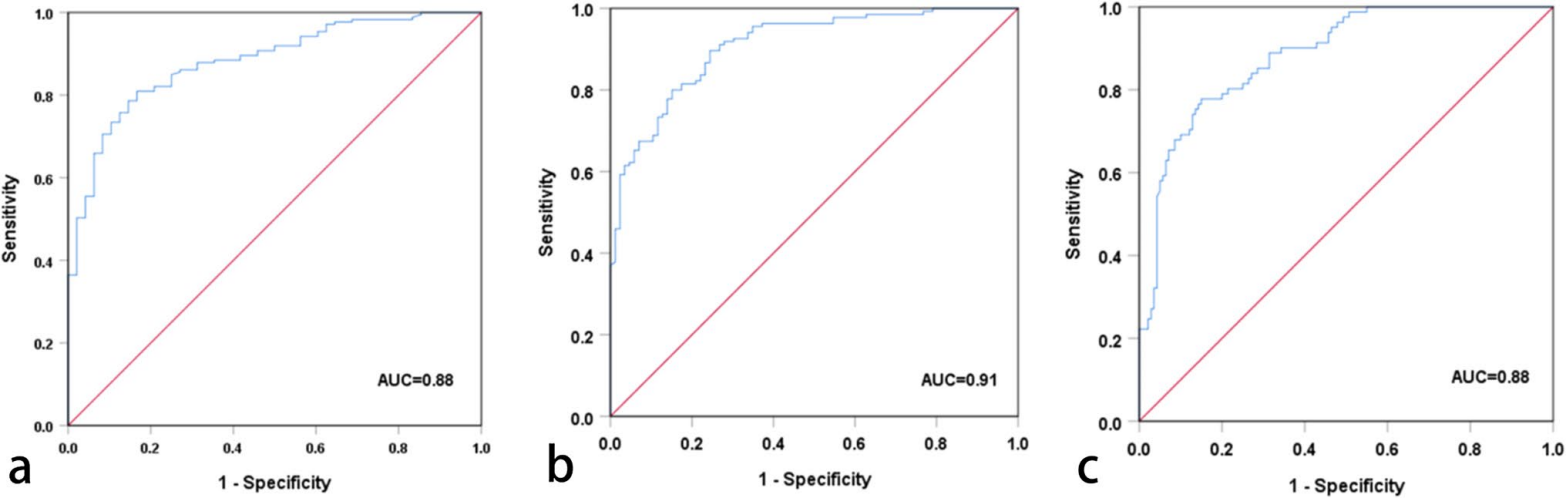
ROC curves of different risk types:(**a**) PC (**b**) IHPC (**c**) PPC

The SUV_max_ and GG were positively correlated in the different risk groups (r = 0.54, P < 0.01) (Fig. 3). A box plot of the SUV_max_ in the different risk groups is shown in Fig. 4. Figure 5 shows the percentage of patients in each group with an SUV_max_ above and below the diagnostic threshold of PC, IHPC and PPC. According to the results of the ROC curve and ^68^Ga-PSMA PET/CT data, the best SUV_max_ thresholds for the diagnosis of intracapsular PC and PPC were 7.95 and 13.94, respectively. The detailed results are shown in Table 3. The ROC curves for the diagnosis of PC, IHPC and PPC are shown in Fig. 6. According to the ROC curve, when the SUV_max_ was 13.94, the sensitivity, specificity, positive predictive value (PPV), negative predictive value (NPV), Youden index, and AUC of ^68^Ga-PSMA PET/CT for diagnosing PPC are 0.78, 0.85, 0.75, 0.87, 0.63, and 0.88, respectively.

**Table 3:** Results of ^68^ Ga-PSMA PET/CT in diagnosing intraprostatic cancers

Groups	Cutoff (SUV _max_)	Sensitivity	Specificity	PPV	NPV	Youden index	AUC
PC	7.95	0.81	0.83	0.95	0.55	0.64	0.88
IHPC	8.54	0.90	0.76	0.85	0.82	0.65	0.91
PPC	13.94	0.78	0.85	0.75	0.87	0.63	0.88

*PC* prostate cancer, *IHPC* intermediate- to high-risk prostate cancer, *PPC* poor prognosis prostate cancer, *SUVmax* maximum standardized uptake value, *PPV* positive predictive value, *NPV* negative predictive value, *AUC* the area under the curve

## Discussion

This study investigated the value of ^68^Ga-PSMA PET/CT for the preoperative diagnosis of intracapsular PPC. This technique can help improve the confidence of radiologists and clinicians in the diagnosis of PPC before radical prostatectomy and thus increase the reliability of delivery to patients. Diagnostic information can help clinicians and patients make the best choice when discussing treatment options [17]. This study showed that the specificity (85%) and NPV (87%) of ^68^Ga-PSMA PET/CT for the diagnosis of PPC in the capsule were relatively high, and the sensitivity (90%) and accuracy (AUC = 0.91) for the diagnosis of IHPC were high. For intracapsular PPC, high specificity and NPV usually mean high clinical value, which can be reflected in the following aspects. When PET/CT imaging is negative for prostate cancer lesions, it is highly reliable in ruling out the presence of patients with metastasis. This can provide more accurate disease staging information, which is crucial for determining the accurate location and extent of tumors. This provides patients with more treatment options and to some extent reduces the occurrence of unnecessary biopsies or chemotherapy, reducing the potential treatment-related complications and adverse effects that patients may face, helping improve their quality of life.

Although the 10-year survival rate of PC is approximately 90%, advanced PC may be life-threatening, especially in the metastatic stage. Mottet N et al. [5] showed that PSA screening alone detected more low-risk PC patients but had no significant effect on PC mortality (median follow-up of 10 years), indicating that these patients may have received unnecessary treatment. TRUS needle biopsy may detect no clinically significant PCs and miss the diagnosis of clinically significant prostate cancers (up to 30%), resulting in imprecise risk stratification. However, compared with TRUS-guided biopsy, mpMRI-targeted biopsy yields 20% more clinically significant prostate cancers [18]. Due to the low specificity of mpMRI, the mpMRI results of low-risk patients might lead to unnecessary biopsies caused by false positives. Currently, mpMRI is not recommended as an initial screening tool before needle biopsy [19].

The diagnostic threshold and the median in the present study were slightly lower than those in our previous study. Previous studies included all PCs, so some of the patients had distant metastasis and extracapsular invasion. These patients had a greater degree of malignancy than the other patients and had no early lesions at the time of diagnosis, so qualitative diagnosis was easier. The main role of the examination was to perform a staging evaluation, but the diagnostic threshold was higher. One of the most important clinical tasks when encountering lesions without extracapsular invasion or distant metastasis is to perform qualitative assessment and predictive risk stratification, which was also the main purpose of this study. We studied intracapsular PC, and easy-to-diagnose intermediate to advanced PC patients were not included. Therefore, the proportion of our patients with low- and intermediate-risk PC with relatively slow progression was greater (approximately 81%). The results of this study differ from those of previous studies [20], whose threshold and median values are higher than us(threshold:8 vs 7.95; median:26.1 vs 13.68). The result of this study can provide guidance for when early intracapsular PC is encountered in clinical practice.

The SUV_max_ is a quantifiable objective indicator and one of the main reference standards for subjective diagnosis. The range of SUV_max_ is 0 to 100. It is difficult to visually detect gray-level differences in images with SUV_max_ above 10; such differences can be detected only through computerized measurements, which are different from those of MR T2W and apparent diffusion coefficient (ADC) images. The gray-level variation range and the difference in the extreme values of the ADC values of the MR images are small, so the differences can be identified visually. Therefore, the magnetic resonance Prostate Imaging-Reporting and Data System (PI-RADS) diagnostic criteria have been recognized worldwide, but the empirical and subjective diagnostic criteria for PSMA-RADS (Prostate-Specific Membrane Antigen Reporting and Data System) reliability still need extensive validation [19, 21]. PSMA-RADS is a standardized system for interpreting ^68^Ga-PSMA PET/CT imaging in patients with prostate cancer. It provides a structured approach to reporting imaging findings, which can help in better interpretation and communication among healthcare providers, but there are some potential shortcomings to consider. Most importantly, it may not be widely adopted or standardized across all institutions. Variability in reporting practices may exist between different institutions, making it challenging to compare results across centers. Though PSMA-RADS provides a structured approach to interpreting ^68^Ga-PSMA PET/CT imaging, its clinical utility may be influenced by potential limitations. Continued research and validation are necessary to further refine and optimize its use in clinical practice [22, 23].This study found that SUV_max_ was positively correlated with PC risk. However, the median SUV_max_ of high-risk PCs was the highest at GG3, whereas the SUV_max_ at GG4 and 5 gradually decreased, which was not the expected increase in SUV_max_ with increasing score. This difference may be related to the greater degree of malignancy of IHPC and the reduced expression level of PSMA.

The aim of the current study was assessment of involved intracapsular lesions using ^68^Ga-PSMA PET/CT imaging. Most of these malignant lesions were still in the early stage of development, and the peak time of their malignant characteristics had not yet been reached. Previous studies [24–26] have fully discussed the diagnostic value of PET/CT for PC patients with metastasis, which is more easily diagnosed in clinical practice. When patients occur metastasis, their prognosis are often poor with limited treatment options and decreased survival rates. Our article mainly discusses the diagnostic value of PET/CT for patients without metastasis, whose malignancy is not as high as those with metastasis, and because there is no metastasis or extracapsular invasion, it is often impossible to have clear qualitative staging results even after receiving MRI examination. These patients require puncture biopsy to determine staging, but this is an invasive examination that can cause certain risks and adverse reactions to patients. PSMA is a highly specific targeted probe, particularly valuable in staging and grading. So this article mainly explores the performance of PET/CT in diagnosing intracapsular PPC, and finds that it has high specificity and NPV, therefore they can be detected early and timely, which will be more beneficial for the prognosis of patients and the selection of clinical plans. This study showed that the reliability of ^68^Ga-PSMA PET/CT for detecting low-risk PC was low; and even though BL and low-risk PC could not be clearly distinguished, the diagnostic reliability of high-risk PC was high [27]. Our study found that PET/CT has a relatively low NPV in diagnosing low-risk PC, resulting in poor diagnostic reliability. This is also the main limitation of ^68^Ga-PSMA PET/CT examination method [28, 29]. Firstly, it depends on the expression level of PSMA, but not all prostate cancer cells are highly expressing PSMA, especially low-grade tumors may have lower PSMA expression. And the spatial resolution of PET/CT imaging is limited, which may result in poor detection performance for small lesions with lower radioactive condensation. Therefore, for small or low metabolic activity prostate cancer lesions, PET/CT imaging may not be able to detect them, leading to an increased false negative rate of negative results. In addition, false positives caused by prostatitis or infection may also be another reason for low NPV although its occurrence rate is relatively low. Inflammatory reactions can increase the metabolic activity of local prostate tissue, leading to an increase of PSMA expression in prostate tissue. This may be mistaken for prostate cancer lesions, resulting in false positive results in PET/CT imaging.

This study once again confirms that SUV_max_ has a high positive predictive value and AUC for the diagnosis of all PCs and PPCs and demonstrated its value for the differentiation of patients with GG2 and GG3 [30]. The SUV_max_ of the GG3 PCs was significantly greater than that of the GG2 PCs and was close to the median of the PPCs. If these GG3 patients are included in the high-risk prostate cancer group, the diagnostic performance of the SUV_max_ could be significantly improved, which again indirectly confirmed the significance of GG3. These patients have a worse than GG2 patients, so we considered GG3 patients to PPC with worse prognosis [31, 32].

Our study has some limitations. The first limitation is that we studied a single-center model, and the reference threshold for the SUV_max_ obtained was not universal. This is also a shortcoming shared by other studies [13, 33]. Because the models used in each research center differ, the diagnostic thresholds for benign and malignant prostate lesions in the published literature are between 4 and 11, and each threshold can be used only as a diagnostic reference for one research center, which is also one of the reasons to establish a PSMA-RADS diagnostic standard. To overcome this limitation, a multicenter study needs to analyze the causes of the differences in the threshold under the conditions of uniform drug concentration and injection volume. Additionally, one of the drawbacks of PET is that its resolution is not as high as MRI's, we do not recommend using PSMA PET as the preferred examination method for primary PC. We prioritize economical and radiation free MRI techniques. But PSMA PET has targeted imaging characteristics that MRI does not have, and can compensate for the shortcomings of MRI. Therefore, in order to detect PPC as soon as possible, we recommend performing PSMA PET examination when PC is suspected but MRI examination is ambiguous.

## Conclusion

^68^Ga-PSMA PET/CT has high specificity and NPV in diagnosing intracapsular PPC, but its sensitivity for diagnosing intracapsular low-risk PC is low, which may cause some early low-risk PCs to be missed.

## Data Availability

The datasets used and/or analyzed during the current study available from the corresponding author on reasonable request.
